# Supplementary material for: Intensive hunting changes human-wildlife relationships
Source: PeerJ. 2022 Oct 11;10:e14159. doi: 10.7717/peerj.14159 (PMC9563281; doi:10.7717/peerj.14159)
Supplement: Supplemental Information 1 — Those species that are heavily managed and hunted are shown in blue, bars show standard error. Hunted species were relatively more abundant with higher occupancy in North Carolina, driven by white-tailed deer. Hunted species (i.e., roe deer) were relatively less abundant with lower occupancy compared to unhunted species (i.e., red fox) in Germany. [file peerj-10-14159-s001.docx]

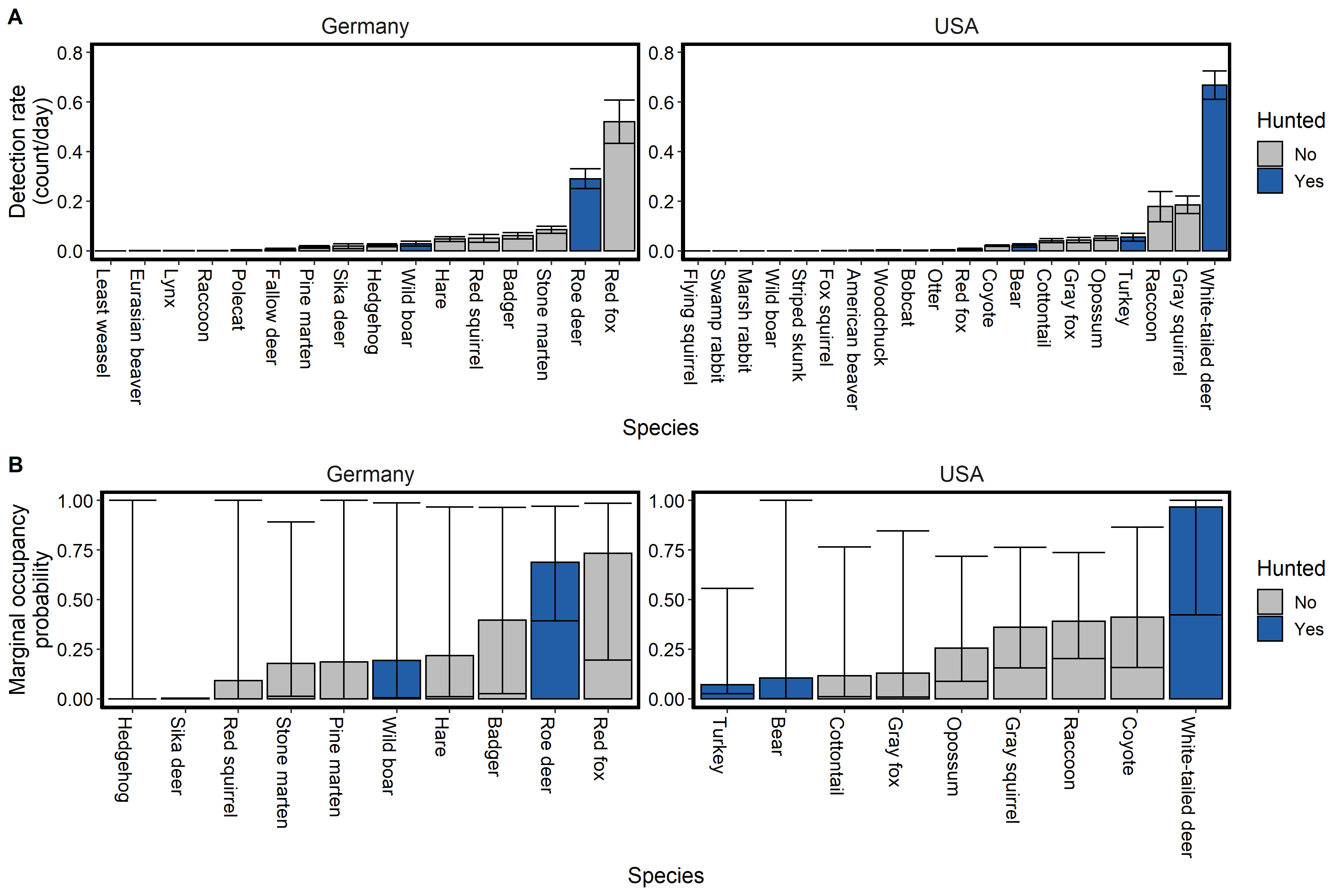
Supplemental Figure S1: Relative abundance (detection rate (count/day); A) and marginal occupancy (B) for mammal species detected on cameras run in Germany and North Carolina, USA. Data are taken from camera traps (242 sites in NC and 233 in BW). Those species that are heavily managed and hunted are shown in blue, bars show standard error. Hunted species were relatively more abundant with higher occupancy in North Carolina, driven by white-tailed deer. Hunted species (i.e., roe deer) were relatively less abundant with lower occupancy compared to unhunted species (i.e., red fox) in Germany.
