# Supplementary material for: Intensive hunting changes human-wildlife relationships
Source: PeerJ. 2022 Oct 11;10:e14159. doi: 10.7717/peerj.14159 (PMC9563281; doi:10.7717/peerj.14159)
Supplement: Supplemental Information 2 — Data are taken from camera traps run in Germany (European roe deer) and North Carolina, USA (white-tailed deer, wild turkey, bear). Those species shown are the only hunted species to have a significant difference in the relationship between time since last human detection and detection intensity between hunted and unhunted areas. White-tailed deer showed evidence of temporal avoidance of humans in hunted areas only while roe deer showed evidence of temporal avoidance of humans in unhunted areas only. Bears showed evidence of temporal attraction to humans in unhunted areas only. Turkeys showed evidence of temporal avoidance of humans in unhunted areas only. Wild boars showed evidence of avoidance of humans in both hunted and unhunted areas, with higher levels of avoidance in hunted areas. [file peerj-10-14159-s002.docx]

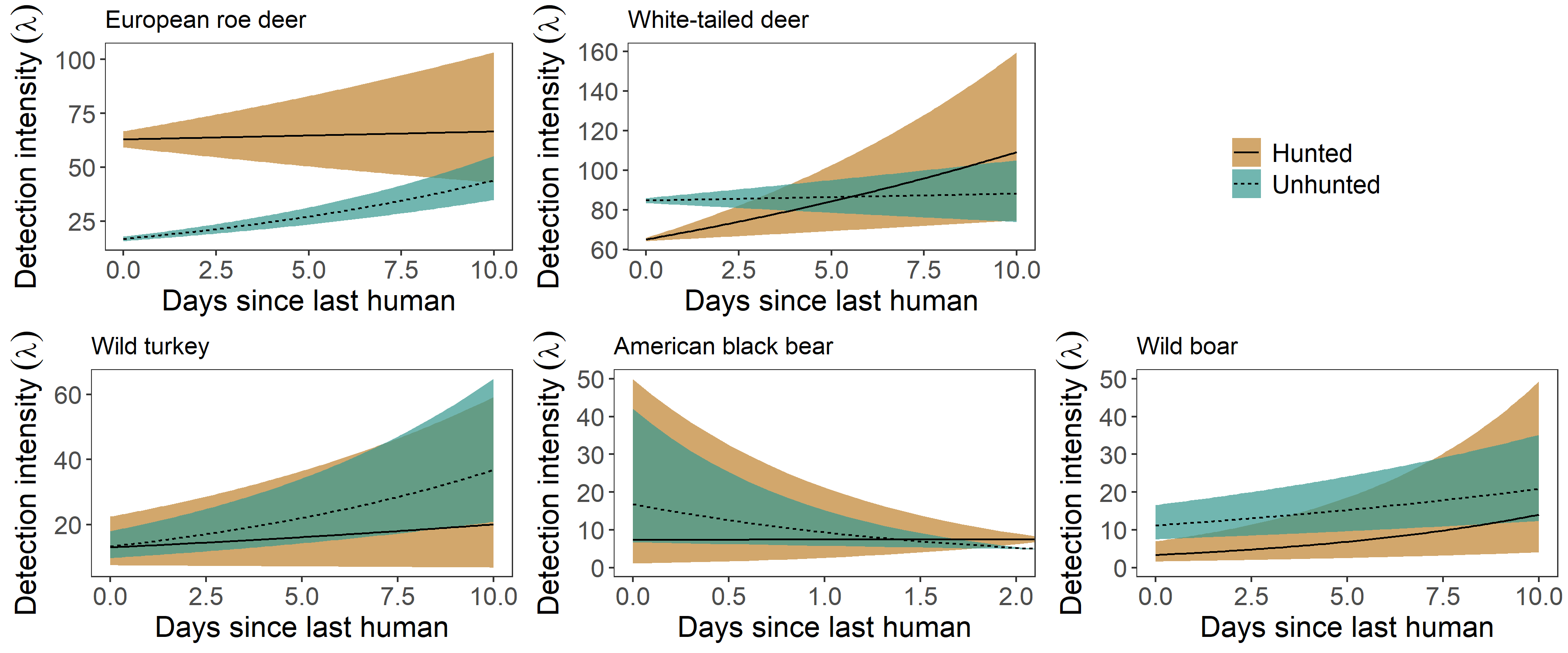


Supplemental Figure S2: Temporal interactions of four hunted wildlife species with humans measured as the effect of days since the last human detection on wildlife detection intensity compared between hunted and unhunted areas. Data are taken from camera traps run in Germany (European roe deer) and North Carolina, USA (white-tailed deer, wild turkey, bear). Those species shown are the only hunted species to have a significant difference in the relationship between time since last human detection and detection intensity between hunted and unhunted areas. White-tailed deer showed evidence of temporal avoidance of humans in hunted areas only while roe deer showed evidence of temporal avoidance of humans in unhunted areas only. Bears showed evidence of temporal attraction to humans in unhunted areas only. Turkeys showed evidence of temporal avoidance of humans in unhunted areas only. Wild boars showed evidence of avoidance of humans in both hunted and unhunted areas, with higher levels of avoidance in hunted areas.
