# Supplementary material for: Intensive hunting changes human-wildlife relationships
Source: PeerJ. 2022 Oct 11;10:e14159. doi: 10.7717/peerj.14159 (PMC9563281; doi:10.7717/peerj.14159)
Supplement: Supplemental Information 3 — Values are given as Germany—USA and values in parentheses show standard error. [file peerj-10-14159-s003.docx]

| Supplemental Table S1: Number of samples (camera trap sites) and average human detection rate (count/day) within three different habitat types (residential yards, open areas and forested areas) between two countries, Germany and the USA. Values are given as Germany\|USA and values in parentheses show standard error. | | |
| --- | --- | --- |
| **Germany\|USA** | **Hunted** | **Unhunted** |
| Yard samples | 0\|33 | 103\|75 |
| Open samples | 0\|30 | 77\|49 |
| Forest samples | 39\|20 | 14\|35 |
| Total samples | 39\|50 | 194\|192 |
| Human detection rate, yard | 0\|0.1(0.04) | 0.63(0.10)\|0.16(0.08) |
| Human detection rate, open | 0\|0.09(0.04) | 0.12(0.02)\|2.49(1.47) |
| Human detection rate, forest | 0.06(0.02)\|0.06(0.04) | 0.03(0.02)\|0.14(0.06) |
| Overall human detection rate | 0.06(0.02)\|0.08(0.02) | 0.39(0.06)\|0.87(0.45) |
