# Supplementary material for: Intensive hunting changes human-wildlife relationships
Source: PeerJ. 2022 Oct 11;10:e14159. doi: 10.7717/peerj.14159 (PMC9563281; doi:10.7717/peerj.14159)
Supplement: Supplemental Information 4 — Data are taken from camera traps run in each country (242 sites in NC and 233 in BW), stratified along an urbanization gradient and among yards, forest fragments and open areas. Species marked with a * are heavily managed and hunted. A temporal relationship that depends on hunting (i.e., a significant interaction effect between time since last human detection and whether an area was hunted) indicates that the temporal relationship with humans is different in hunted and unhunted areas. [file peerj-10-14159-s004.docx]

| Supplemental Table S2: Results of a multispecies continuous-time occupancy model examining simultaneous co-occurrence (spatial relationship) and co-detection (temporal relationship) between wildlife species and humans in two countries, Germany and North Carolina, USA. Data are taken from camera traps run in each country (242 sites in NC and 233 in BW), stratified along an urbanization gradient and among yards, forest fragments and open areas. Species marked with a * are heavily managed and hunted. A temporal relationship that depends on hunting (i.e., a significant interaction effect between time since last human detection and whether an area was hunted) indicates that the temporal relationship with humans is different in hunted and unhunted areas. | | | | | |
| --- | --- | --- | --- | --- | --- |
| Species | Country | Number detections | Spatial relationship with humans | Temporal relationship with humans | Temporal effect magnitude |
| White-tailed deer* | USA | 3322 | None | Depends on hunting | 0.012 |
| Eastern gray squirrel | USA | 913 | None | Depends on hunting | -0.020 |
| Northern raccoon | USA | 796 | None | Depends on hunting | -0.009 |
| Virginia opossum | USA | 254 | None | Avoidance | 0.060 |
| Wild turkey* | USA | 252 | None | Depends on hunting | 0.300 |
| Eastern cottontail | USA | 215 | Same sites | None | 0.021 |
| Gray fox | USA | 210 | Same sites | Avoidance | 0.104 |
| Coyote | USA | 116 | Area_of_nearest_urban mediated | Avoidance | 0.085 |
| American black bear* | USA | 108 | None | Depends on hunting | -1.720 |
| Red fox | Germany | 3752 | Same sites | Avoidance | 0.121 |
| European roe deer* | Germany | 2522 | None | Depends on hunting | 0.282 |
| Stone marten | Germany | 661 | None | Avoidance | 0.115 |
| Eurasian badger | Germany | 424 | Same sites | Avoidance | 0.088 |
| Eurasian red squirrel | Germany | 391 | None | None | 0.002 |
| European hare | Germany | 304 | None | Depends on hunting | 0.076 |
| Wild boar* | Germany | 235 | None | Depends on hunting | 0.183 |
| Western European hedgehog | Germany | 174 | None | Avoidance | 0.086 |
| Sika deer | Germany | 146 | None | None | 0.003 |
| European pine marten | Germany | 103 | None | Depends on hunting | 0.277 |
