# Supplementary material for: Intensive hunting changes human-wildlife relationships
Source: PeerJ. 2022 Oct 11;10:e14159. doi: 10.7717/peerj.14159 (PMC9563281; doi:10.7717/peerj.14159)
Supplement: Supplemental Information 5 — For occupancy, each covariate is categorized by whether it was used in the spatial model, temporal model or both. [file peerj-10-14159-s005.docx]

| Supplemental Table S3: Covariates used in the occupancy and detection rate analyses. For occupancy, each covariate is categorized by whether it was used in the spatial model, temporal model or both. | | | | |
| --- | --- | --- | --- | --- |
| Interaction Type | Covariate | Description | Type | Model |
| Spatial | Forest | % forested in 1km radius^1^ | GIS | ψ, DR |
| Spatial | Urban | % urban in 1km radius^1^ | GIS | ψ, DR |
| Spatial | Forest*Urban | Interaction term between forest and urban landcovers | Interaction | ψ, DR |
| Spatial | Urban_area | Size of closest urban area (km^2^) | GIS | ψ, DR |
| Spatial | Urban_dist | Distance to closest urban area (km) | GIS | ψ, DR |
| Spatial | YardYN | 0/1 indicator of whether site is residential yard | Camera site | Ψ, DR |
| Spatial and Temporal | Hunt | 0/1 indicator of whether site is hunted | Camera site | Ψ, DR, p |
| Temporal | TSH | Time elapsed since the last detection of human at the site (days) | Temporal and camera site | p |
| Temporal | TSH*Hunt | Interaction of hunting and time elapsed since last detection of a human at the site | Interaction | p |
| 1. A global map of terrestrial habitat types (Jung *et al.* 2020) | | | | |
|  | | | | |
|  | | |  |  |
