# Supplementary material for: Intensive hunting changes human-wildlife relationships
Source: PeerJ. 2022 Oct 11;10:e14159. doi: 10.7717/peerj.14159 (PMC9563281; doi:10.7717/peerj.14159)
Supplement: Supplemental Information 6 — We calculated the relative ecological impact of each species, specific to three trophic levels (plants, invertebrates, vertebrates), by accounting for their metabolically active mass and diet. Below are specific reference we used as source material to parameterize this analysis. [file peerj-10-14159-s006.docx]

Supplemental Table S4: Resources for calculating ecological impacts. We calculated the relative ecological impact of each species, specific to three trophic levels (plants, invertebrates, vertebrates), by accounting for their metabolically active mass and diet. Below are specific reference we used as source material to parameterize this analysis.

| **Parameter** | **Source** |
| --- | --- |
| **Metabolically active mass** | Ramirez, J.I., Jansen, P.A., den Ouden, J., Li, X., Iacobelli, P., Herdoiza, N. & Poorter, L. (2021) Temperate forests respond in a non-linear way to a population gradient of wild deer. *Forestry: An International Journal of Forest Research***,** In Press. |
| **Species-specific diet, Turkey** | Dunning, J.B.J. (2008) *CRC Handbook of Avian Body Masses, 2nd Edition*. CRC Press.  Glover, F.A. & Bailey, R.W. (1949) Wild turkey foods in West Virginia. *The Journal of Wildlife Management,* **13,** 255-265. |
| **Species-specific diet, Mammals** | Soria, C.D., Pacifici, M., Di Marco, M., Stephen, S.M. & Rondinini, C. (2021) COMBINE: a coalesced mammal database of intrinsic and extrinsic traits. |
