# Supplementary material for: Intensive hunting changes human-wildlife relationships
Source: PeerJ. 2022 Oct 11;10:e14159. doi: 10.7717/peerj.14159 (PMC9563281; doi:10.7717/peerj.14159)
Supplement: Supplemental Information 7 — Data are taken from camera traps run in each country (sampled 242 sites in NC and 233 in BW), stratified along an urbanization gradient and among yards, forest fragments and open areas. Species marked with a * are heavily managed and hunted. Coefficients marked with a R and O were statistically different from 0 in detection rate and occupancy models, respectively. [file peerj-10-14159-s007.docx]

| Supplemental Table S5: Coefficients associated with predictors of detection rate and marginal occupancy for species detected in Germany and the United States. Data are taken from camera traps run in each country (sampled 242 sites in NC and 233 in BW), stratified along an urbanization gradient and among yards, forest fragments and open areas. Species marked with a * are heavily managed and hunted. Coefficients marked with a R and O were statistically different from 0 in detection rate and occupancy models, respectively. | | | | | | |
| --- | --- | --- | --- | --- | --- | --- |
| Species | Yard (Y/N) | Percent urban in a 1km radius | Percent forest in a 1km radius | % urban x % forest | Area (km2) of nearest urban | Distance to nearest urban area |
| **Germany** | | | | | | |
| Eurasian badger |  | +R,O | +R,O | +R |  |  |
| Eurasian red squirrel |  |  | +O |  |  |  |
| European hare | -R,O | -R,O | -R,O | -R,O |  |  |
| European pine marten |  |  | +R |  | +R,O |  |
| European roe deer* | -R,O | -R |  |  |  | +O |
| Red fox |  |  |  |  |  |  |
| Sika deer |  |  |  |  | +O |  |
| Stone marten | +R,O |  |  |  |  |  |
| W. European hedgehog | +R |  |  |  | +O |  |
| Wild boar* | -R |  |  |  |  | +R,O |
|  |  |  |  |  |  |  |
| **USA** | | | | | | |
| Bear* |  |  |  |  |  | +R,O |
| Coyote |  |  |  |  |  |  |
| Eastern cottontail |  |  |  |  |  |  |
| Eastern gray squirrel | +R | +R,O |  |  |  | -R |
| Gray fox |  |  |  |  |  | -R |
| Northern raccoon |  | +R,O |  |  |  |  |
| Virginia opossum | +O |  |  |  |  |  |
| White-tailed deer* | -O |  |  |  |  |  |
| Wild turkey* |  | -R,O |  |  |  |  |
