# Supplementary material for: Intensive hunting changes human-wildlife relationships
Source: PeerJ. 2022 Oct 11;10:e14159. doi: 10.7717/peerj.14159 (PMC9563281; doi:10.7717/peerj.14159)
Supplement: Supplemental Information 8 — Parentheses show SE. [file peerj-10-14159-s008.docx]

| Supplemental Table S6: Mean detection rates (count/day) for each species in each country compared between hunted and unhunted sites and forests, open areas and residential yards. Data are taken from camera traps run in Germany and North Carolina, USA. Parentheses show SE. | | | | | | | |
| --- | --- | --- | --- | --- | --- | --- | --- |
|  |  | **Hunted** | | | **Unhunted** | | |
| Species | Country | Forest | Open | Yard | Forest | Open | Yard |
| American black bear | USA | 0.07 (0.04) | 0.09 (0.04) | 0.01 (0.01) | 0.01 (0.01) | 0.01 (0.01) | 0 (0) |
| Coyote | USA | 0.02 (0.01) | 0.02 (0.01) | 0.03 (0.01) | 0.02 (0.01) | 0.02 (0.01) | 0.02 (0.01) |
| Eastern cottontail | USA | 0.03 (0.02) | 0.01 (0.01) | 0.07 (0.03) | 0.02 (0.01) | 0.05 (0.02) | 0.03 (0.01) |
| Eastern gray squirrel | USA | 0.02 (0.01) | 0.01 (0) | 0.21 (0.11) | 0.21 (0.1) | 0.1 (0.03) | 0.32 (0.08) |
| Gray fox | USA | 0.02 (0.01) | 0.04 (0.03) | 0.03 (0.01) | 0.07 (0.05) | 0.04 (0.01) | 0.05 (0.02) |
| Northern raccoon | USA | 0.14 (0.08) | 0.03 (0.02) | 0.06 (0.02) | 0.19 (0.12) | 0.04 (0.01) | 0.24 (0.13) |
| Virginia opossum | USA | 0.03 (0.01) | 0.02 (0.01) | 0.07 (0.03) | 0.02 (0.01) | 0.05 (0.02) | 0.06 (0.01) |
| White-tailed deer | USA | 0.46 (0.11) | 0.82 (0.18) | 0.66 (0.14) | 0.75 (0.16) | 0.55 (0.07) | 0.59 (0.1) |
| Wild turkey | USA | 0.02 (0.01) | 0.06 (0.03) | 0.04 (0.02) | 0.04 (0.03) | 0.02 (0.01) | 0.07 (0.04) |
| Domestic cat | USA | 0.01 (0) | 0 (0) | 0.13 (0.1) | 0.02 (0.01) | 0.02 (0.01) | 0.05 (0.02) |
| Eurasian Badger | Germany | 0.04 (0.02) |  |  | 0.1 (0.04) | 0.03 (0.01) | 0.08 (0.03) |
| Eurasian Red Squirrel | Germany | 0.06 (0.03) |  |  | 0.16 (0.12) | 0.01 (0.01) | 0.07 (0.03) |
| European Hare | Germany | 0.06 (0.02) |  |  | 0.06 (0.03) | 0.1 (0.03) | 0 (0) |
| European Pine Marten | Germany | 0.06 (0.02) |  |  | 0.04 (0.02) | 0 (0) | 0 (0) |
| European Roe Deer | Germany | 0.78 (0.17) |  |  | 0.4 (0.08) | 0.32 (0.05) | 0.03 (0.02) |
| Red Fox | Germany | 0.14 (0.03) |  |  | 0.34 (0.1) | 0.37 (0.07) | 0.73 (0.18) |
| Sika Deer | Germany | 0 (0) |  |  | 0.28 (0.15) | 0.01 (0) | 0 (0) |
| Stone Marten | Germany | 0.01 (0) |  |  | 0.01 (0.01) | 0.02 (0) | 0.17 (0.03) |
| Western European Hedgehog | Germany | 0 (0) |  |  | 0 (0) | 0 (0) | 0.04 (0.01) |
| Wild Boar | Germany | 0.04 (0.02) |  |  | 0.08 (0.03) | 0.05 (0.02) | 0 (0) |
| Domestic cat | Germany | 0.01 (0) |  |  | 0.08 (0.03) | 0.07 (0.03) | 1.38 (0.21) |
